# Serum uric acid and prognosis in acute ischemic stroke: a dose–response meta-analysis of cohort studies

**DOI:** 10.3389/fnagi.2023.1223015

**Published:** 2023-09-01

**Authors:** Wenyuan Zhang, Zicheng Cheng, Fangwang Fu, Zhenxiang Zhan

**Affiliations:** ^1^Department of Neurology, Affiliated Yueqing Hospital of Wenzhou Medical University, Yueqing, China; ^2^Department of Neurology, Affiliated Jinhua Hospital, Zhejiang University School of Medicine, Jinhua, China; ^3^Department of Neurology, The Second Affiliated Hospital and Yuying Children’s Hospital of Wenzhou Medical University, Wenzhou, China

**Keywords:** acute ischemic stroke, dose–response meta-analysis, functional outcome, prognosis, uric acid

## Abstract

**Background:**

There have been contradictory findings regarding the relationship between serum uric acid levels and prognosis in acute ischemic stroke. Whether this association is nonlinear due to uric acid’s paradoxical properties (antioxidant and prooxidant) is unclear.

**Methods:**

We searched PubMed, Web of Science, and Embase databases until December 2022. Cohort studies reporting serum uric acid levels and functional outcome, mortality, or neurological complications in patients with acute ischemic stroke were included. Summary effect estimates were calculated using a random-effect model. Moreover, dose–response relationships were assessed by the generalized least squares trend estimation.

**Results:**

Altogether, 13 cohort studies were identified in this study. Compared to the lowest baseline serum uric acid levels, the highest levels were associated with decreased risk of poor functional outcome (OR = 0.70, 95% CI 0.54–0.91, *I*^2^ = 29%), hemorrhagic transformation (OR = 0.15, 95% CI 0.05–0.42, *I*^2^ = 79%), and post-stroke depression (OR = 0.04, 95% CI 0.00–0.95, *I*^2^ = 89%), but not associated with mortality and symptomatic intracerebral hemorrhage. A nonlinear relationship was observed in poor functional outcome (U-shaped, *P* for nonlinearity = 0.042), hemorrhagic transformation (inverse, *P* for nonlinearity = 0.001), and post-stroke depression (inverse, *P* for nonlinearity = 0.002). In addition, there was a single study reporting a U-shaped association in post-stroke epilepsy (*P* for nonlinearity <0.001). Furthermore, another study reported a positive curvilinear association in stroke recurrence (*P* for nonlinearity <0.05). The insufficient number of original articles for some prognostic indicators should be considered when interpreting the results of this meta-analysis.

**Conclusion:**

In patients with acute ischemic stroke, serum uric acid levels are nonlinearly associated with the risk of poor functional outcome (U-shaped). More evidence is needed to confirm the association between serum uric acid levels and neurological complications following acute ischemic stroke.

## Introduction

Approximately 950 individuals per 100,000 population suffer from acute ischemic stroke worldwide (Collaborators G.B.D.S, 2021). Only half of the patients can perform their usual duties and activities, and up to 10% die 90 days after stroke onset (Wang et al., 2022). In addition to the direct neurological impairment caused by cerebral infarction, neurological complications, such as hemorrhagic transformation, seizures, and depression, lead to delays in rehabilitation, prolonged hospital stays, poor functional outcomes, and increased cost of care (Balami et al., 2011). Although early reperfusion therapy has improved the adverse outcome of acute ischemic stroke (Goyal et al., 2016), the high disability and mortality rates of the disease require more effective therapies (e.g., neuroprotection).

Uric acid is an abundant and potent endogenous antioxidant that effectively scavenges reactive nitrogen and oxygen radicals (Gulcin et al., 2008). Longitudinal studies confirmed that high serum uric acid levels at baseline are associated with decreased risk of functional dependence in patients with acute ischemic stroke (Lee et al., 2014; Wang et al., 2018). However, uric acid has a prooxidative property and is a double-edged sword for humans (Sautin and Johnson, 2008). Thus, many studies found a nonlinear relationship between serum uric acid levels and functional outcome (Seet et al., 2010; Zhang et al., 2016; Yang et al., 2018) or neurological complications (Wang et al., 2019; Li et al., 2020; Yuan et al., 2020) in acute ischemic stroke. A recent meta-analysis showed no significant associations of serum uric acid levels with functional outcome and mortality in acute ischemic stroke (Zhang et al., 2021). However, this meta-analysis did not consider the dual antioxidative and prooxidative properties of uric acid and dose–response analysis was not performed to determine whether a nonlinear relationship existed.

In a randomized, double-blind phase 2b/3 trial, the authors found that adding uric acid to thrombolytic therapy in acute ischemic stroke patients had an insignificant effect on the increase of excellent outcome (OR = 1.23, 95% CI 0.96–1.56). This could be due to the potential nonlinear relationship between serum uric acid levels and functional outcome in acute ischemic stroke, resulting in an optimal node for serum uric acid levels. Some recent studies have added evidence to the associations of uric acid with functional outcome (Bai et al., 2022; Liu et al., 2022a,b), mortality (Bai et al., 2022; Liu et al., 2022a), hemorrhagic transformation (Cheng et al., 2022; Tian et al., 2022), and symptomatic intracerebral hemorrhage (Bai et al., 2022; Cheng et al., 2022). Thus, this dose–response meta-analysis of cohort studies aimed to update and clarify the shape of the relationship between serum uric acid levels and prognosis in patients with acute ischemic stroke.

## Methods

This meta-analysis was performed and reported following a predefined protocol (PROSPERO registration number: CRD42022384410) and the Preferred Reporting Items for Systematic Reviews and Meta-Analyses (PRISMA) guidelines (Page et al., 2021).

### Search strategy

We conducted a systematic literature search using PubMed, Web of Science, and Embase databases from inception to December 2022. The following search strategy was used: (“uric acid” OR “urate”) AND (“stroke” OR “cerebral infarction” OR “brain infarction”). We also manually searched relevant articles’ references to identify additional eligible studies.

### Study selection

Studies were included in this meta-analysis if they met the following inclusion criteria: (1) the study reported the associations between serum uric acid levels and prognosis in patients with acute ischemic stroke; (2) the study design was a cohort study; (3) the concentrations of serum uric acid were required and divided into ≥3 groups of different levels; (4) the study outcomes were functional outcome, mortality, or neurological complications; (5) effect estimates and corresponding 95% confidence intervals (CIs) were reported; and (6) event size and sample size information was clear. The following studies were excluded: (1) the study design was a cross-sectional or case–control study; (2) enrolled participants were hemorrhagic stroke or mixed stroke; (3) effect estimates were not accessible; and (4) only abstracts were available. When there was only one eligible article for a particular outcome measure, the article was not included in the meta-analysis. The articles not eligible for meta-analysis were included in the systematic review when nonlinear relationships between serum uric acid levels and outcome measures were tested.

The prognostic indicators included functional outcome, mortality, and neurological complications. Functional outcome was usually evaluated using the modified Rankin Scale (mRS), and an unfavorable functional outcome was defined as mRS > 1 or 2. Neurological complications, including hemorrhagic transformation, symptomatic intracerebral hemorrhage, epilepsy, affective diseases, and recurrent stroke, were considered.

A prearranged procedure for selecting eligible literature was independently performed by two investigators (WZ and ZC). Potential studies were screened by reviewing titles and abstracts, and full texts were checked for eligibility according to the inclusion and exclusion criteria. In cases of disagreement, a third reviewer (FF) joined the discussion.

### Data extraction

Two independent investigators (WZ and ZC) collected data using a standard electronic form. The following information was extracted from each included study: first author’s last name, publication year, country, study design, sample size, age at baseline, percentage of females, serum uric acid levels, follow-up duration, outcome assessment, number of events, and adjusted confounders. In addition, we extracted the number of events and sample and effect estimates of the different exposure categories. The effect estimates from the most fully adjusted model were selected from the studies reporting several multivariable-adjusted effect estimates.

### Quality assessment

The cohort subscale of the Newcastle-Ottawa Scale (NOS) was used to evaluate the risk of bias in the study design (Stang, 2010). The scale consists of three domains: selection, comparability, and outcome. The quality score ranges from 0 to 9 points, and a score ≥ 7 indicates a high-quality study. Two independent reviewers (WZ and ZC) used the scale to calculate the total score and any discrepancies were resolved by a third reviewer (FF).

### Statistical analysis

We calculated summary ORs of the serum uric acid levels (the highest vs. the lowest) and each prognostic indicator using the random-effects model (DerSimonian and Laird method) (DerSimonian and Laird, 1986). The log-transformed ORs and 95% CIs from each study were pooled to calculate the pooled estimates. We conducted a dose–response meta-analysis to calculate pooled estimates from the natural logarithm of ORs and 95% CIs across categories of serum uric acid levels. For each category of serum uric acid, we used the midpoint of serum uric acid levels if the mean or median level per category was not reported. When extreme categories were open-ended, we used the width of the adjacent interval to calculate an upper or lower cut-off value. The method described by Greenland and Longnecker was used for linear dose–response analysis (Greenland and Longnecker, 1992). Nonlinear dose–response analysis was performed using restricted cubic splines with three knots at 10, 50, and 90% percentiles of the distribution (Orsini et al., 2012). A heterogeneity test was performed employing the Cochran Q statistic and *I*^2^ metric; *I*^2^ > 50% was considered statistically significant heterogeneity (Higgins et al., 2003). All statistical tests were two-sided, and *p* < 0.05 was considered statistically significant. Statistical analyses were performed using R version 4.2.1 software (R Foundation for Statistical Computing, Vienna, Austria).

## Results

### Literature search

**Figure 1:**
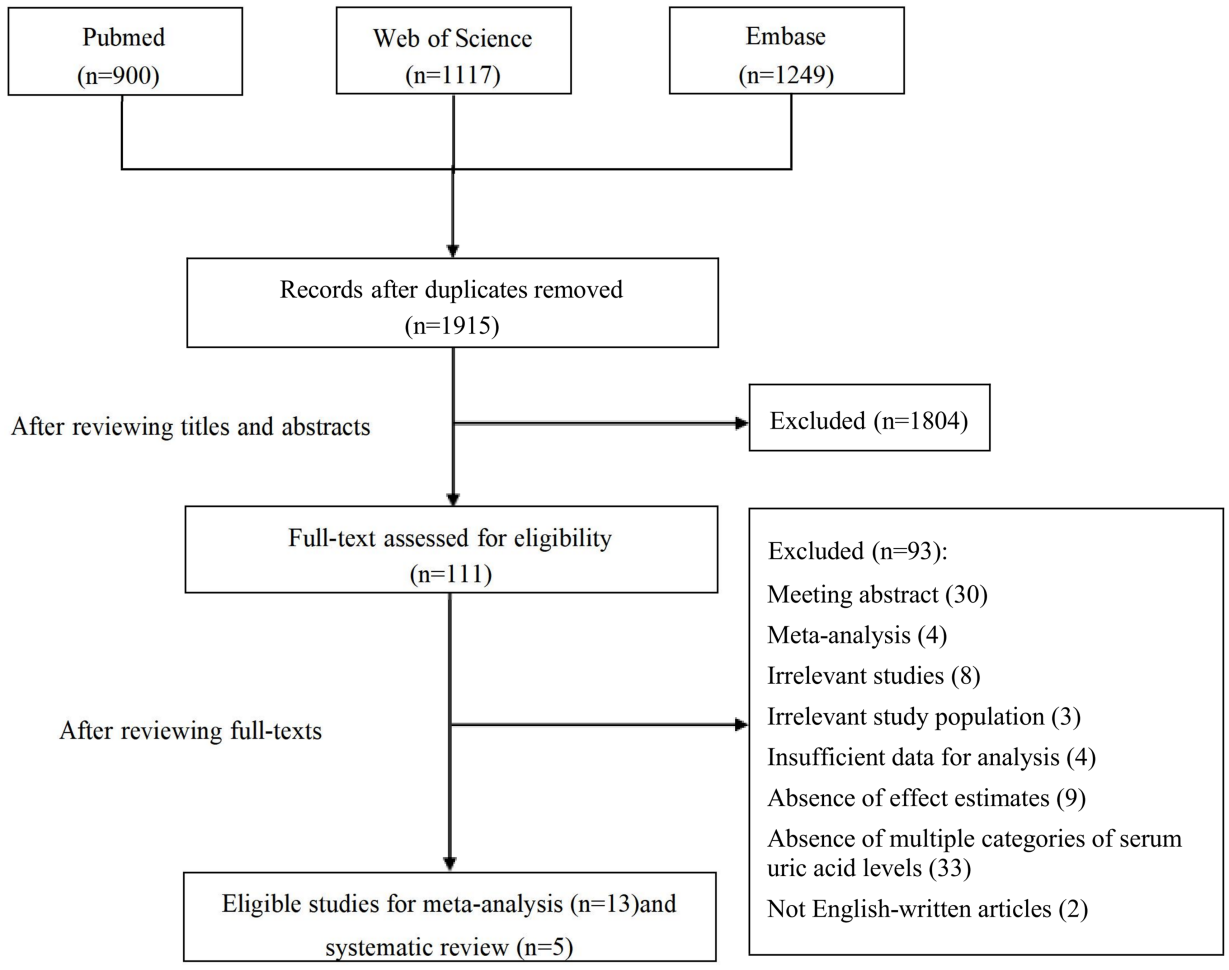
Study flowchart.

Overall, 1915 articles were identified from the initial database search after deduplication. The study selection process is shown in Figure 1. After the initial screening based on titles and abstracts, 111 studies were retained for further evaluation following the full text read. Subsequent to detailed evaluations, 13 studies were finally included in this meta-analysis, and another five studies were elaborated on the systematic review. A manual search of the reference lists of these studies did not yield any new eligible studies.

### Study characteristics

The characteristics of the included studies for meta-analysis are presented in Table 1. Almost all studies were conducted in China. Eight studies were prospective cohorts, whereas five were retrospective cohorts. Six of the included studies were restricted to patients undergoing intravenous thrombolysis or endovascular thrombectomy. A total of 10,485 participants (median sample size *n* = 611; minimum *n* = 196 and maximum *n* = 3,370) were enrolled in these studies. The mean age of the patients was between 37.8 and 70.6 years, and the proportion of females ranged from 15.6–56.9%. The mean serum uric acid levels of the patients were between 288.5 and 348.6 μmol/L. Outcome measures comprised functional outcome (Lee et al., 2014; Zhang et al., 2016; Wang et al., 2018; Chen et al., 2020; Yuan et al., 2020; Bai et al., 2022; Liu et al., 2022a,b), mortality (Wang et al., 2018; Chen et al., 2020; Bai et al., 2022; Liu et al., 2022a), hemorrhagic transformation (Chen et al., 2020; Song et al., 2020; Cheng et al., 2022; Tian et al., 2022), symptomatic intracerebral hemorrhage (Lee et al., 2014; Chen et al., 2020; Song et al., 2020; Yuan et al., 2020; Bai et al., 2022; Cheng et al., 2022), and post-stroke depression (Gu et al., 2015; Li et al., 2020). Follow-up intervals ranged from 14 days to 12 months for functional outcome and mortality, 36 h to 7 days for hemorrhagic transformation, 24 h to 7 days for symptomatic intracerebral hemorrhage, and 9 days to 3 months for post-stroke depression. According to the quality assessment using NOS (Table 2), nine studies scored ≥7.

**Table 1:** Study characteristics.

First author, year	Country	Study design	Study subjects	Sample size	Age (mean, y)	Female	Serum uric acid levels (mean, μmol/L)	Midpoint of serum uric acid levels in groups with the highest and lowest levels (μmol/L)	Outcome	Outcome assessment	Number of events	Follow-up time	Adjusted confounders	NOS
Lee, 2014	Korea	Prospective	Intravenous thrombolysis	218	67.6 ± 10.3	56.9%	NA	345, 214	Favorable functional outcome	mRS = 0 when baseline NIHSS <8, mRS < 2 when baseline NIHSS = 8–14, and mRS < 3 when baseline NIHSS >14	62 (28.4%)	3 months	None	4
									Symptomatic intracerebral hemorrhage	ECASS II criteria	15 (6.9%)	48 h	None	
Gu, 2015	China	Prospective	General	196	61.2 ± 9.9	33.7%	288.5 ± 62.4	373, 195	Post-stroke depression	DSM-IV criteria	56 (28.6%)	3 months	Age, sex, NIHSS	9
Zhang, 2016	China	Prospective	General	303	64.6 ± 12.8	33.7%	319.9 ± 89.9	413, 218	Poor functional outcome	mRS > 1	107 (35.3%)	3 months	Age, sex, comorbidities, medications, NIHSS	7
Wang, 2018	China	Retrospective	General	1,166	64.5 ± 13.4	37.3%	305.0 ± 103.1	408, 208.5	Poor functional outcome	mRS > 1	294 (32.9%)	12 months	Age, sex, comorbidities	5
									Mortality	mRS = 6	35 (3.0%)	12 months	None	
Chen, 2020	China	Retrospective	Endovascular thrombectomy	247	63.1 ± 12.6	27.2%	340.0 ± 97.4	436, 236.5	Poor functional outcome	mRS > 2	162 (65.6%)	3 months	None	7
									Mortality	mRS = 6	29 (11.8%)	3 months	None	
									Hemorrhagic transformation	ECASS II criteria	92 (37.2%)	72 h	Age, sex, comorbidities, medications	
									Symptomatic intracerebral hemorrhage	ECASS II criteria	22 (8.9%)	72 h	None	
Li, 2020	China	Prospective	General	498	57.2 ± 10.9	23.9%	348.6 ± 106.6	437, 255	Post-stroke depression	DSM-IV criteria	232 (46.6%)	9 days	Age, sex, comorbidities, NIHSS	7
Song, 2020	China	Prospective	General	1,230	64.1 ± 14.5	36.5%	342.4 ± 105.2	420, 250	Hemorrhagic transformation	ECASS II criteria	133 (10.8%)	7 days	Age, sex, comorbidities, medications, NIHSS	9
									Symptomatic intracerebral hemorrhage	NINDS criteria	21 (1.7%)	7 days	Age, sex, comorbidities, medications, NIHSS	
Yuan, 2020	China	Prospective	Endovascular thrombectomy	611	64.7 ± 12.3	40.1%	318.2 ± 100.8	416, 216.5	Favorable functional outcome	mRS < 3	262 (42.9%)	3 months	None	6
									Symptomatic intracerebral hemorrhage	HBC criteria	90 (14.7%)	72 h	Age, sex, comorbidities	
Bai, 2022	China	Prospective	Endovascular thrombectomy	780	64.4 ± 12.1	33.7%	310.0 ± 96.2	398, 219.5	Poor functional outcome	mRS > 1	550 (70.5%)	3 months	Age, sex, comorbidities, NIHSS	6
									Mortality	mRS = 6	144 (18.5%)	3 months	None	
									Symptomatic intracerebral hemorrhage	ECASS II criteria	47 (6.0%)	24 h	None	
Cheng, 2022	China	Retrospective	Intravenous thrombolysis	503	69.3 ± 13.4	34.6%	316.3 ± 97.4	399.5, 221	Hemorrhagic transformation	ECASS II criteria	60 (11.9%)	36 h	Age, sex, NIHSS	8
									Symptomatic intracerebral hemorrhage	NINDS criteria	22 (4.4%)	36 h	Age, sex, NIHSS	
Liu, 2022 (1)	China	Retrospective	General	3,370	70.6 ± 13.6	42.9%	315.4 ± 101.2	404, 208	Poor functional outcome	mRS > 2	1,579 (46.9%)	14 days	None	7
									Mortality	mRS = 6	133 (3.9%)	14 days	None	
Liu, 2022 (2)	China	Prospective	General	636	37.8 ± 6.8	15.6%	NA	457.5, 259.5	Poor functional outcome	mRS > 1	154 (24.2%)	3 months	Age, sex, comorbidities, NIHSS	9
Tian, 2022	China	Retrospective	Intravenous thrombolysis	727	64.1 ± 11.9	30.0%	306.4 ± 96.6	389.5, 209.5	Hemorrhagic transformation	ECASS II criteria	112 (15.4%)	7 days	Age, sex, comorbidities, NIHSS	7

ECASS, European Cooperative Acute Stroke Study; HBC, Heidelberg Bleeding Classification; mRS, modified Rankin Scale; NA, not applicable; NIHSS, National Institutes of Health Stroke Scale; NINDS, National Institute of Neurological Disorders and Stroke; NOS, Newcastle-Ottawa Scale.

**Table 2:** The quality assessment of studies by the Newcastle Ottawa scale.

Study	Selection items				Comparability items		Outcome items			Total score
	Representativeness of exposed cohort	Representativeness of unexposed cohort	Ascertainment of exposure	Outcome not present at start of study	Age	Stroke severity	Assessment of outcome	Follow-up length	Adequacy of follow-up	
Lee, 2014	0	1	1	0	0	0	0	1	1	4
Gu, 2015	1	1	1	1	1	1	1	1	1	9
Zhang, 2016	1	1	1	0	1	1	0	1	1	7
Wang, 2018	1	1	0	0	1	0	0	1	1	5
Chen, 2020	0	1	1	1	1	1	0	1	1	7
Li, 2020	1	1	0	1	1	1	1	0	1	7
Song, 2020	1	1	1	1	1	1	1	1	1	9
Yuan, 2020	0	1	0	1	1	0	1	1	1	6
Bai, 2022	0	1	0	1	1	1	0	1	1	6
Cheng, 2022	0	1	1	1	1	1	1	1	1	8
Liu, 2022 (1)	1	1	1	0	1	1	1	0	1	7
Liu, 2022 (2)	1	1	1	1	1	1	1	1	1	9
Tian, 2022	0	1	0	1	1	1	1	1	1	7

### Associations of uric acid with functional outcome and mortality

**Figure 2:**
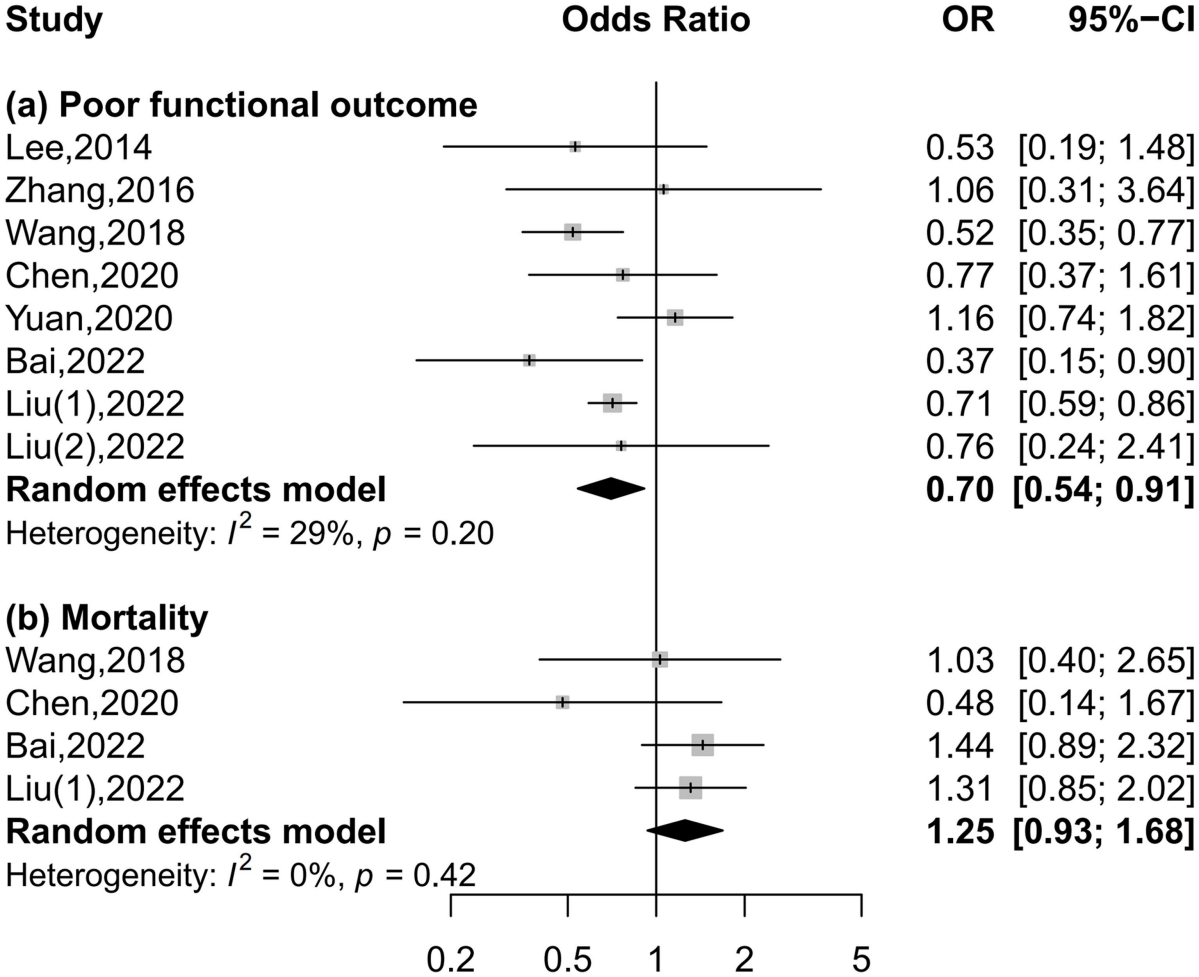
Forest plots depicting the association of serum uric acid levels with poor functional outcome and mortality in acute ischemic stroke.

**Figure 3:**
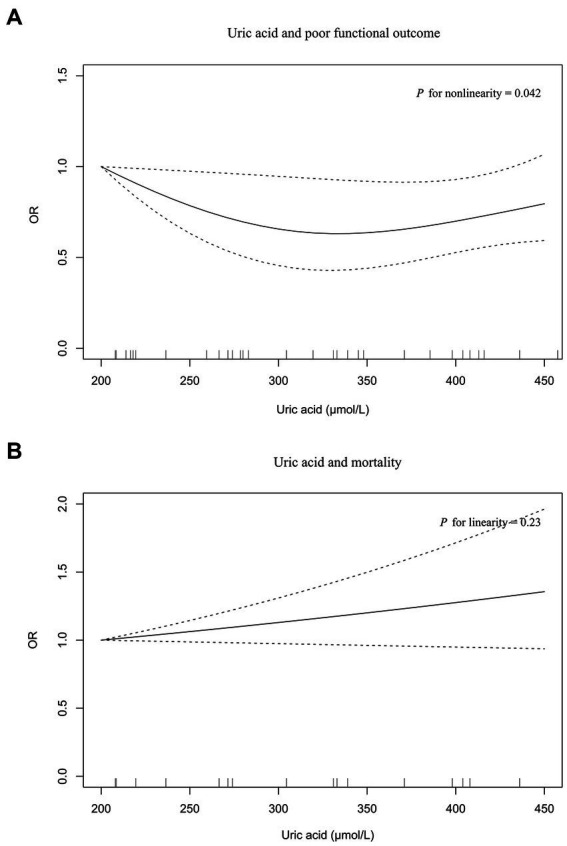
The dose–response plot on the association of serum uric acid levels with poor functional outcome **(A)** and mortality **(B)** in acute ischemic stroke.

Eight and four studies were included in the meta-analysis on functional outcome and mortality, respectively (Figure 2). There was a 30% reduction in the risk of poor functional outcome in patients with the highest serum uric acid levels than those with the lowest levels (OR = 0.70, 95% CI 0.54–0.91, *I*^2^ = 29%). The risk of mortality was equivalent between patients with the highest and lowest serum uric acid levels (OR = 1.25, 95% CI 0.93–1.68, *I*^2^ = 0%). Dose–response meta-analysis revealed a U-shaped association between serum uric acid levels and poor functional outcome (*P* for nonlinearity = 0.042, Figure 3A). The risk of poor functional outcome fell until around 330 μmol/L of serum uric acid levels and increased afterward. No association between serum uric acid levels and mortality was observed in the dose–response meta-analysis (*P* for linearity = 0.23, Figure 3B). According to the study design, serum uric acid levels were not associated with poor functional outcome in prospective studies, but U-shaped association was still present in retrospective studies (Supplementary Figures S1–S3).

### Association of uric acid with neurological complications

**Figure 4:**
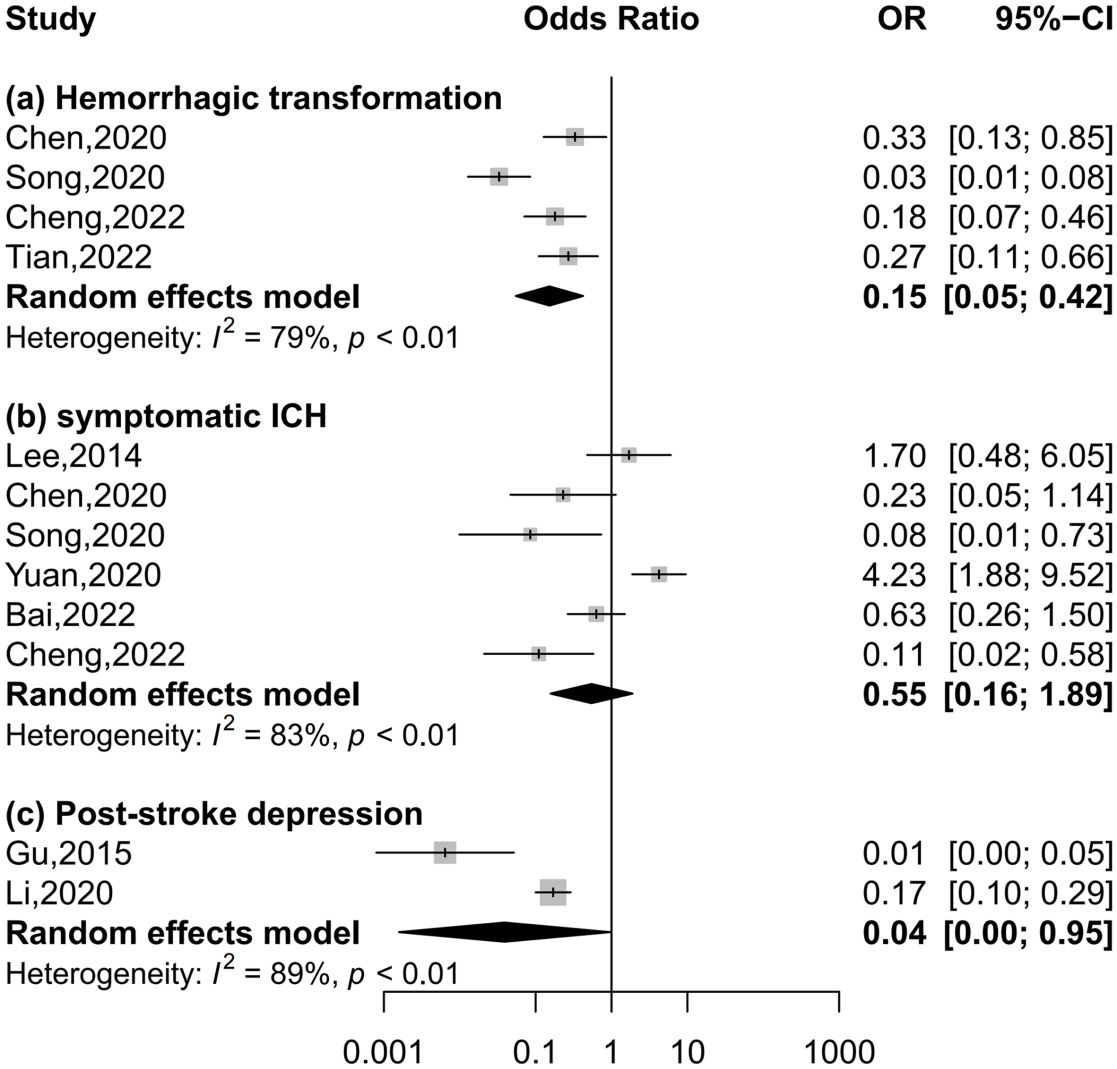
Forest plots depicting the association of serum uric acid levels with neurological complications in acute ischemic stroke.

**Figure 5:**
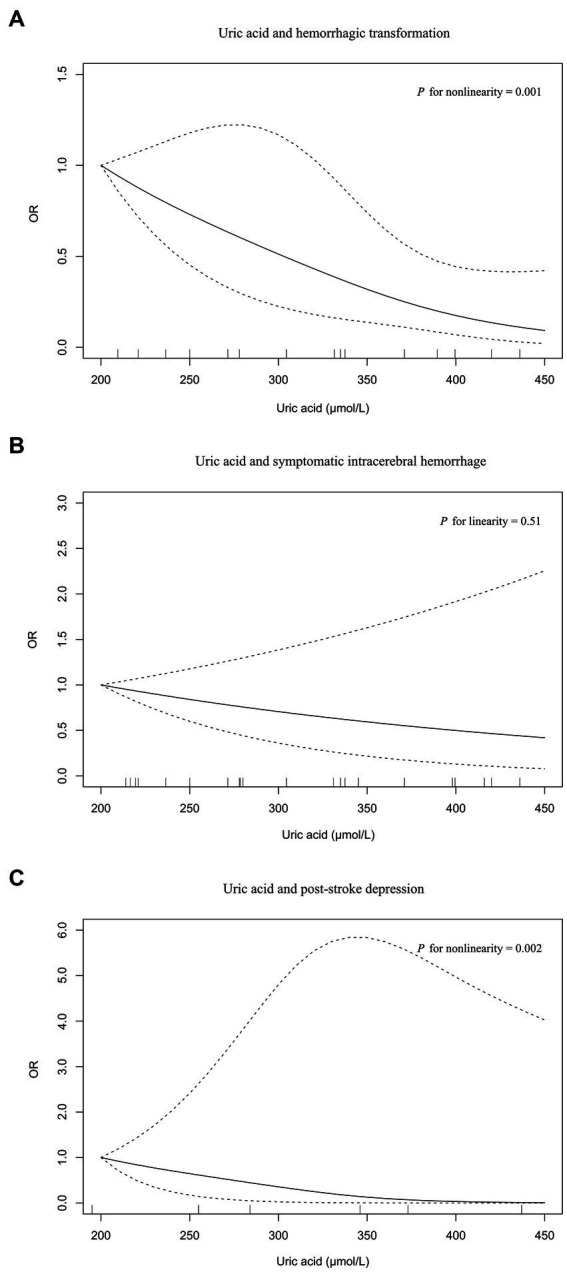
The dose–response plot on the association of serum uric acid levels with neurological complications in acute ischemic stroke: **(A)** hemorrhagic transformation, **(B)** symptomatic intracerebral hemorrhage, **(C)** post-stroke depression.

Four, six, and two studies were included in the meta-analysis on hemorrhagic transformation, symptomatic intracerebral hemorrhage, and post-stroke depression, respectively (Figure 4). Compared to patients with the lowest serum uric acid levels, patients with the highest levels had a significantly lower risk of hemorrhagic transformation (OR = 0.15, 95% CI 0.05–0.42, *I*^2^ = 79%) and post-stroke depression (OR = 0.04, 95% CI 0.00–0.95, *I*^2^ = 89%) and equal risk of symptomatic intracerebral hemorrhage (OR = 0.55, 95% CI 0.16–1.89, *I*^2^ = 83%). Nonlinear relationships of serum uric acid levels with hemorrhagic transformation (*P* for nonlinearity = 0.001, Figure 5A) and post-stroke depression (*P* for nonlinearity = 0.002, Figure 5C) were observed, and a maximum reduction in risk for both when the levels were up to 400–450 μmol/L. There was no evidence of any link between serum uric acid levels and symptomatic intracerebral hemorrhage based on the dose–response meta-analysis (*P* for linearity = 0.51, Figure 5B).

### Systematic review

We further conducted a systematic review of the literature, not eligible for meta-analysis, examining nonlinear relationships between serum uric acid levels and prognosis in patients with acute ischemic stroke.

In a prospective study of transient ischemic attack and ischemic stroke, patients with serum uric acid levels between 340 and 410 μmol/L had the lowest risk of poor functional outcome at 12 months compared to those with levels <340 and > 410 μmol/L (Seet et al., 2010). In another population of first-ever ischemic stroke, the risk of poor functional outcome was lowest in patients with serum uric acid levels of the 60–70 percentile (corresponding to 286–324 μmol/L) (Yang et al., 2018). However, a large sample cohort enrolling 2,564 patients with normal renal function and elevated systolic blood pressure showed a linear association between serum uric acid levels and poor functional outcome (*P* for nonlinearity = 0.60, *P* for linearity = 0.023) (Zheng et al., 2020). A cross-sectional study compared serum uric acid levels in patients with acute ischemic stroke, post-stroke epilepsy, and post-stroke status epilepticus and found a U-shaped dose-effect relationship of serum uric acid levels with post-stroke epilepsy (*P* for nonlinearity <0.001) and post-stroke status epilepticus (*P* for nonlinearity <0.001) (Wang et al., 2019). Recently, Zhu et al. demonstrated that serum uric acid levels were nonlinearly associated with ischemic stroke recurrence (Zhu et al., 2022). The risk of stroke recurrence increased rapidly with a rise in serum uric acid levels to 300 μmol/L, then slowed down.

## Discussion

To our knowledge, this is the first meta-analysis about the potential nonlinear effects of serum uric acid levels on the prognosis of acute ischemic stroke. Based on the effect estimates reported in included studies, we found decreased risk of poor functional outcome, hemorrhagic transformation, and post-stroke depression in patients with the highest serum uric acid levels than those with the lowest levels. Nonlinear relationships were observed in functional outcome, hemorrhagic transformation, post-stroke depression, post-stroke epilepsy, post-stroke status epilepticus, and stroke recurrence. Serum uric acid levels were not associated with the risk of mortality and symptomatic intracerebral hemorrhage.

Wang and colleagues first performed a meta-analysis including 10 studies with 8,131 acute ischemic stroke patients (Wang et al., 2016). Compared to low serum uric acid levels, high serum uric acid levels were associated with decreased risk of poor outcome (HR = 0.77, 95% CI 0.68–0.88). Patients with good outcomes had higher serum uric acid levels than those with poor outcomes (mean difference = 30.6 μmol/L, 95% CI 20.13–41.08). Later, Lei et al. added more studies and updated the meta-analysis, yielding similar results (Lei et al., 2019). However, a recent meta-analysis comprising 10 studies with 9,632 acute ischemic stroke patients revealed no significant associations of serum uric acid levels with functional outcome (OR = 0.99, 95% CI 0.97–1.10), poor outcome (OR = 1.07, 95% CI 0.99–1.15), vascular events (OR = 0.86, 95% CI 0.52–1.41), and mortality (OR = 1.08, 95% CI 0.93–1.24). The above three meta-analyses did not consider the potential nonlinear relationship between serum uric acid levels and prognosis. Our meta-analysis has several advantages over earlier reports. First, a dose–response meta-analysis was adopted to confirm the nonlinear relationship between serum uric acid levels and poor functional outcome. Second, the association between serum uric acid levels and neurologic complications was examined by meta-analysis for the first time. Third, most of the included studies were of high quality and properly adjusted for confounders such as age, sex, stroke severity, medications, and vascular risk factors.

As the end product of purine metabolism, the physiological concentration of serum uric acid in healthy adults is 1.5–6.0 mg/dL for women and 2.5–7.0 mg/dL (1 mg/dL = 59.5 μmol/L) for men (Maiuolo et al., 2016). The neuroprotective properties of uric acid include the capacity to scavenge free radicals generated by the breakdown of peroxynitrite (Squadrito et al., 2000), promoting neuronal glutathione synthesis (Aoyama et al., 2011). Animal models of ischemic stroke indicated that uric acid administration reduced infarct size, blood–brain barrier impairment, brain edema, and neurofunctional deficit (Aliena-Valero et al., 2021). Pilot studies found that uric acid treatment decreased serum oxidative stress markers levels (matrix metalloproteinase-9 and malondialdehyde) and prevented an early fall of serum uric acid levels in acute ischemic stroke patients treated with alteplase (Amaro et al., 2007, 2009). Serial measurement of serum uric acid exposed that most patients had a dynamic decrease in serum uric acid levels during the acute stage of ischemic stroke (Brouns et al., 2010; Wang et al., 2021). The decline in serum uric acid levels from admission to day seven was significantly greater in patients with high National Institutes of Health Stroke Scale (NIHSS) scores, large infarct volume, or poor functional outcome in a cohort of 199 patients with acute ischemic stroke (*n* = 156) or transient ischemic attack (*n* = 43) (Brouns et al., 2010). Another cohort of 361 acute ischemic stroke patients undergoing reperfusion therapy reported a positive association between decreased serum uric acid levels during hospitalization and the risk of poor functional outcome (Wang et al., 2021). These studies provided insights that higher serum uric acid levels in the early stage might have a protective effect on neurological outcome in patients with acute ischemic stroke.

However, the antioxidant function of uric acid has its limitations; uric acid cannot scavenge superoxide, ascorbic acid and thiols in the plasma are required for the antioxidative effect of uric acid, and uric acid is an antioxidant only in the hydrophilic environment (Sautin and Johnson, 2008). Instead, uric acid can become a prooxidant by forming radicals in reactions with oxidants reactions or engaging intracellular oxidant production *via* the ubiquitous NADPH oxidase-dependent pathway resulting in oxidative stress (Sautin and Johnson, 2008). The paradoxical properties of uric acid can explain previous inconsistent findings on the impact of serum uric acid levels on the prognosis in acute ischemic stroke, including positive, negative and null associations. The paradoxical properties also support our findings that serum uric acid levels are nonlinearly related to prognosis in acute ischemic stroke. A nonlinear relationship between serum uric acid levels and the risk of stroke was revealed in a dose–response meta-analysis comprising 19 prospective cohort studies, consistent with our findings (Qiao et al., 2021). Although subgroup analysis showed that nonlinear relationship between serum uric acid levels and poor functional outcome was present in retrospective studies rather than prospective studies, particularly retrospective studies may be more prone to bias, it should be pointed out that prospective studies have a smaller sample size.

In the URICO-ICTUS study, 1,000 mg uric acid was dissolved in 500 mL of vehicle made of 0·1% lithium carbonate and 5% mannitol and infused intravenously in 90 min during the infusion of alteplase (Chamorro et al., 2014). The pharmacokinetics study showed a mean of peak serum uric levels reaching 10 mg/dL when the infusion was finished, and an increment of serum uric acid levels remained above baseline levels for approximately 24 h (elimination half-life of 44 h) (Amaro et al., 2007). Uric acid treatment increased the proportion of patients with an excellent outcome at 90 days compared with placebo (39% vs. 33%, *p* = 0.099), although the difference was insignificant (Chamorro et al., 2014). We proposed several hypotheses about this marginal effect. On the one hand, the dosing regimen may need further refinement, including dose and duration. Based on our study, the optimal serum uric acid level to obtain favored functional outcome was 330 μmol/L. Observational studies showed persistent serum uric acid level decrement during the first week following stroke onset (Brouns et al., 2010). Thus, a smoother and more lasting dosing regimen can be designed. On the other hand, a combined infusion of other antioxidants (e.g., ascorbic acid) may improve the antioxidative capacity of uric acid.

Although nonlinear associations of serum uric acid levels with poor functional outcome and post-epilepsy were U-shaped, a maximal reduction in the risk of hemorrhagic transformation and post-stroke depression was observed at higher serum uric acid levels. Therefore, optimal serum uric acid levels must be individualized based on the patient’s risk for disability and post-stroke epilepsy or risk for hemorrhagic transformation and post-stroke depression. For stroke recurrence, low serum uric acid levels were associated with a substantial reduction in risk, whereas high serum uric acid levels were affiliated with risk elevation. This positive association represented the prooxidant property of uric acid, consistent with previous findings that a J-shaped association exists between serum uric acid levels and the risk of first stroke (Qiao et al., 2021). Based on the different effects of serum uric acid levels on poor functional outcome and stroke recurrence, the reasonable strategy is to maintain high serum uric acid levels in the acute phase of stroke and low serum uric acid levels after the acute phase.

Several limitations should be noted in our study. First, the number of articles for meta-analysis was limited; therefore, subgroup analysis and publication bias tests were not further performed. The potential source of heterogeneity in the meta-analysis on neurological complications was unclear. Some studies with null results might have remained unpublished, resulting in exaggerated risk estimates. Second, effect estimates were not adjusted for important confounders (Age, sex, comorbidities, medications, and NIHSS score) in about half of the studies. An insignificant risk factor in univariate analysis may actually be a significant risk factor in multivariable analysis if confounding is properly controlled (Sun et al., 1996). Thus, the association between serum uric acid levels and prognosis might have been misjudged in some studies. Third, we could not conduct subgroup analyses based on gender because the original articles lacked the necessary data. Serum uric acid levels are higher in males than in females due to the effect of sex hormones; the reanalysis of the URICO-ICTUS study found that uric acid treatment doubled the rate of excellent outcome after acute ischemic stroke in women (OR = 2.09, 95% CI 1.05–4.15) but not in men (OR = 1.00, 95% CI 0.52–1.93) (Llull et al., 2015). It is necessary to investigate the relationship between serum uric acid levels and prognosis by stratifying analysis of sex. The optimal serum uric acid levels for men and women may differ. Fourth, none of the eligible studies reported the associations between serum uric acid levels and other neurological complications, such as brain edema, sleep disorders, and cognitive impairment. More high-quality longitudinal studies exploring the above association are warranted.

## Conclusion

Serum uric acid levels are inversely associated with the risk of poor functional outcome, hemorrhagic transformation, and post-depression, according to the findings of the current studies. Nonlinear relationships are found between serum uric acid levels and the risk of poor functional outcome (U-shaped), post-epilepsy (U-shaped), hemorrhagic transformation (inverse), post-depression (inverse), and stroke recurrence (positive). Further randomized, controlled trials are warranted to explore whether maintaining serum uric acid at an appropriately higher level during the acute phase improves functional outcome in patients with acute ischemic stroke.

## Data availability statement

The raw data supporting the conclusions of this article will be made available by the authors, without undue reservation.

## Author contributions

WZ and ZC conceived and designed the study. WZ, ZC, and FF performed the literature search and data extraction. ZZ assisted in data analysis. WZ wrote the manuscript. All authors reviewed the manuscript.

## Funding

This work was supported by the Medical Science and Technology Project of Zhejiang Province of China (No. 2021KY797).

## Conflict of interest

The authors declare that the research was conducted in the absence of any commercial or financial relationships that could be construed as a potential conflict of interest.

## Publisher’s note

All claims expressed in this article are solely those of the authors and do not necessarily represent those of their affiliated organizations, or those of the publisher, the editors and the reviewers. Any product that may be evaluated in this article, or claim that may be made by its manufacturer, is not guaranteed or endorsed by the publisher.
