# Supplementary material for: Serum uric acid and prognosis in acute ischemic stroke: a dose–response meta-analysis of cohort studies
Source: Front Aging Neurosci. 2023 Sep 1;15:1223015. doi: 10.3389/fnagi.2023.1223015 (PMC10505709; doi:10.3389/fnagi.2023.1223015)
Supplement: Supplementary file 1 [file Data_Sheet_1.docx]

**Supplemental Material**

Figure S1. Forest plots depicting the association between serum uric acid levels and poor functional outcome in acute ischemic stroke stratified by study design.

Figure S2. The dose-response plot on the association between serum uric acid levels and poor functional outcome in acute ischemic stroke in prospective studies.

Figure S3. The dose-response plot on the association between serum uric acid levels and poor functional outcome in acute ischemic stroke in retrospective studies.


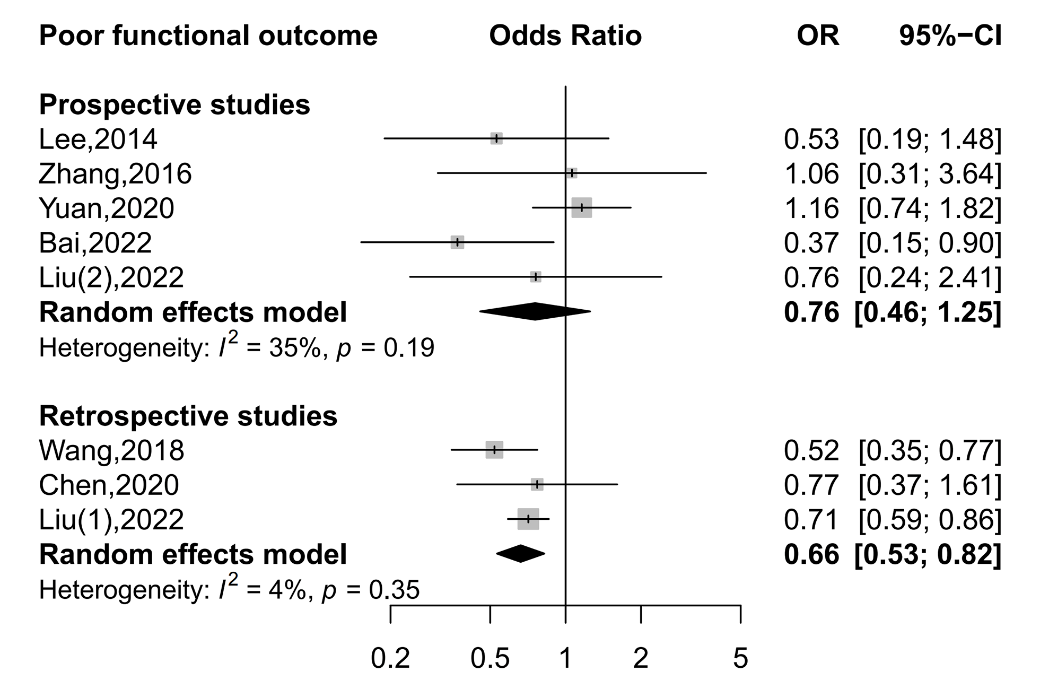


Figure S1. Forest plots depicting the association between serum uric acid levels and poor functional outcome in acute ischemic stroke stratified by study design.


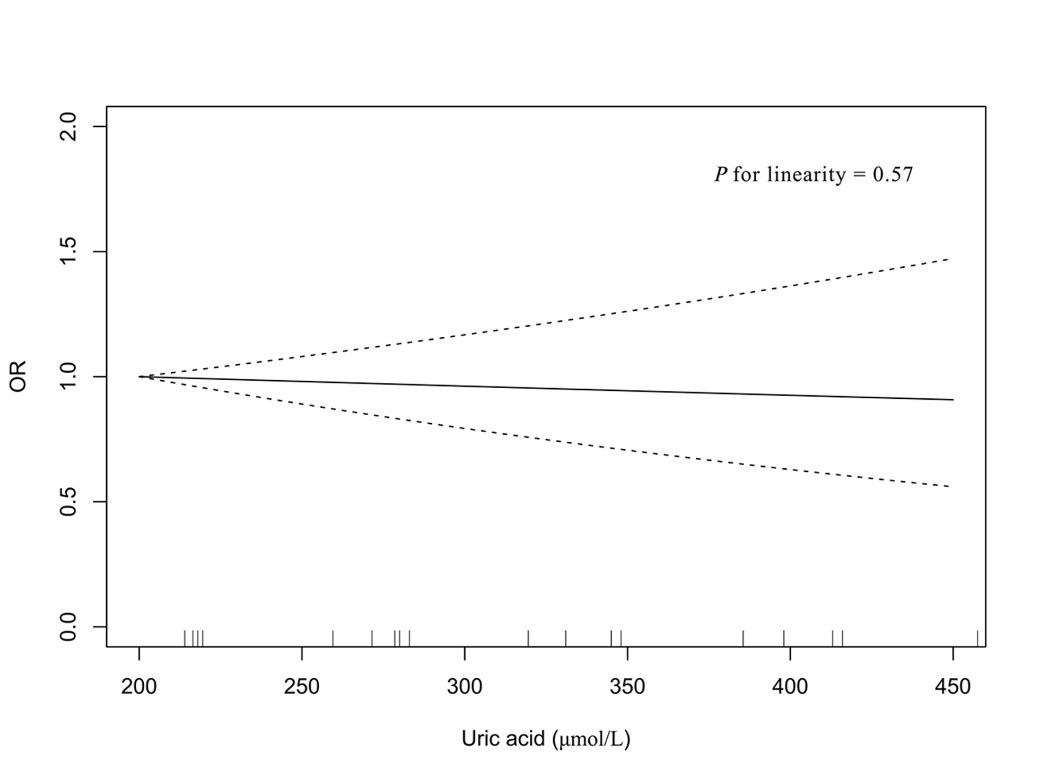


Figure S2. The dose-response plot on the association between serum uric acid levels and poor functional outcome in acute ischemic stroke in prospective studies.


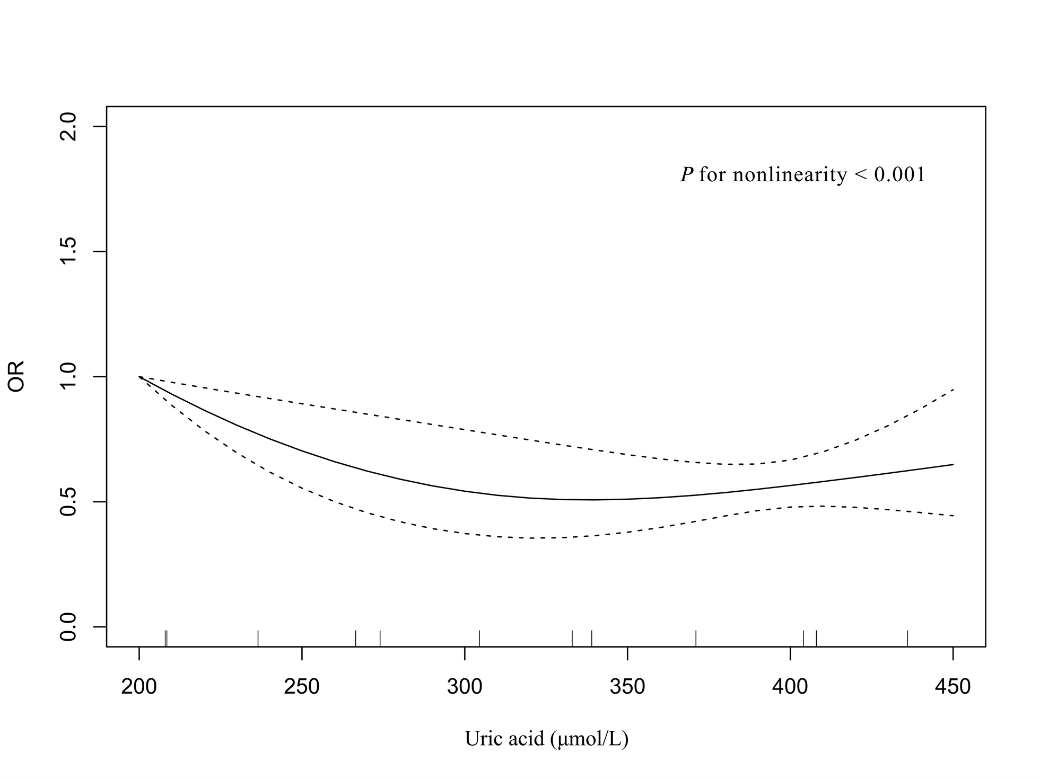


Figure S3. The dose-response plot on the association between serum uric acid levels and poor functional outcome in acute ischemic stroke in retrospective studies.
